# *Centrosomal protein72* rs924607 and vincristine-induced neuropathy in pediatric acute lymphocytic leukemia: meta-analysis

**DOI:** 10.2144/fsoa-2020-0044

**Published:** 2020-05-27

**Authors:** Aida Zečkanović, Janez Jazbec, Marko Kavčič

**Affiliations:** 1 Department for Pediatric Hematology & Oncology, University Children’s Hospital of Ljubljana, University Medical Centre Ljubljana, Bohoričeva street 20, Ljubljana, Slovenia

**Keywords:** acute lymphoblastic leukemia, drug toxicity, peripheral neuropathies, pharmacogenetics, precision medicine, vincristine

## Abstract

**Aim:**

We examined the utility of the rs924607 TT genotype of the *centrosomal protein 72* (*CEP72*) as a potential biomarker for predilection toward vincristine-induced peripheral neuropathy in children treated for acute lymphoblastic leukemia.

**Materials & methods:**

We conducted a random-effects meta-analysis of data from four studies comprising 817 patients. We tested for an association using a recessive model where a one-sided p-value < 0.05 was considered statistically significant.

**Results & conclusion:**

We were unable to confirm the association between the rs924607 TT genotype and neurotoxicity (odds ratio: 1.99; p = 0.16; 95% CI: 0.76–5.25) in our global meta-analysis. Analysis of the continuation phase (following induction) studies showed significantly higher odds for neuropathy in *CEP72* rs924607 TT homozygotes (odds ratio: 2.28; p = 0.02; 95% CI: 1.16–6.87).

## Lay abstract

We analyzed the findings of four previous studies to find out if a variant of a gene important for in cell division called *centrosomal protein 72* is associated with more neurological toxicity in children treated with vincristine for acute lymphoblastic leukemia. We found that this variant was indeed connected with higher odds for developing peripheral neuropathy in the later stages of therapy.

Despite great advances achieved in the last few decades, cancer remains the leading cause of child death by disease in developed countries. Acute lymphoblastic leukemia (ALL) is the predominant childhood malignancy, representing more than 30% of all childhood cancers and approximately 80% of all childhood leukemias. Fortunately, its cure rates have surpassed 85% [1,2]. As more children are cured it is becoming increasingly important to reduce acute and long-term side effects of treatment.

The efficacy of current ALL treatment has been achieved mainly by optimizing the use of existing combinations of chemotherapeutics, rather than adding novel drugs [2]. Therefore, all new information about pharmacokinetics and pharmacogenomics of drugs used in ALL therapy is highly beneficial [3].

Vincristine (VCR) is a very widely used anticancer drug, utilized in pediatric treatment protocols for leukemia as well as solid tumors [2]. It is a vinca alkaloid that causes a mitotic arrest leading to cell death in metaphase. This effect is achieved by disrupting the formation of mitotic spindle microtubules [4]. The major dose-limiting side effect is vincristine-induced peripheral neuropathy (VIPN) which may cause morbidity, necessitate a decrease of VCR dose and thus compromise the effectiveness of treatment [5]. Almost 80% of patients develop VIPN during the treatment and it may affect the quality of their life years after the completion of ALL therapy [6–8]. Presently, we do not have reliable biomarkers to identify patients with predilection toward VIPN.

Several studies have assessed various candidate genes involved in VCR metabolism; however, they have not uncovered genetic variants consistently linked with a higher risk of VIPN [9–20]. The major focus of our article is a gene called centrosomal protein 72 (*CEP72*); an overview of other potential biomarkers is given in Table 1.

**Table 1.** An overview of genes other than *CEP72* that have been reported as potential biomarkers for vincristine-induced peripheral neuropathy.

Gene	Protein function	Variants	Association with VIPN	Ref.
*CYP3A5*	Monooxygenase involved in VCR metabolism. The expressors have a fivefold higher intrinsic VCR clearance than nonexpressors	CYP3A5*1 allele produces an active enzyme. Variants CYP3A5*3, CYP3A5*6 and CYP3A5*7 result in little or no functional enzyme	Expressers of *CYP3A5* have lower VIPN incidence (p = 0.03), lower neurotoxycicty grade and shorter neurotoxicty duration (p = 0.035 and 0.0007, respectively). Ceppi *et al.* did not confirm the association of *CYP3A5* rs776746 (*1/*3) with VIPN Aplenc *et al.* found that CYP3A5*1 was associated with a higher grade of VIPN Franca *et al.* did not confirm the involvement of *CYP3A5* in VCR toxicity	[13,16–18,21,22]
*ACTG1*	Part of the cytoskeleton	rs1135989 allele A	Predilection toward high-grade VCR neurotoxicity (OR: 2.6; 95% CI: 1.1–6.0)	[17]
*ABCB1*	The transport of various molecules across membranes and it is also involved in multidrug resistance	rs4728709 genotype T	A protective effect against low-grade neurotoxicity (OR: 0.3; 95% CI: 0.1–0.9)	[17]
		rs3770102 genotype A	A protective effect against high-grade neurotoxicity (OR: 0.07, 95% CI: 0.01–0.6)	[17]
		Position 1236, 2677 and 3435	No effect on VIPN in children treated with VCR for solid tumors.	[19]
*ABCC1*	The transport of various molecules across membranes and it is also involved in multidrug resistance	Homozygous rs246240 minor allele G	Associated with the onset of grade III/IV neurological toxicity in the induction phase of the AIEOP-BFM ALL 2000 study protocol (OR: 4.61; 95% CI: 1.12–19.02)	[18]
		Rs3784867 genotype TT	Associated with higher incidence of VIPN (OR: 4.91; 95% CI: 1.99–12.10)	[11]
*ABCC2*	The transport of various molecules across membranes and it is also involved in multidrug resistance	rs3740066 GG and rs12826 GG genotypes	Associated with increased neurotoxicity	[14]
*SYNE2*	A nuclear outer membrane protein	rs2781377 genotype AA	Increased risk for VIPN (OR: 2.5; 95% CI: 1.2–5.2)	[15]
*MRPL47*	A component of mitochondrial ribosomes	rs10513762 genotype TT	Increased risk for VIPN (OR: 3.3; 95% CI: 1.4–7.7)	[15]
*BAHD1*	A heterochromatin protein that acts as a transcription repressor	rs3803357 genotype AA	Potentially protective against VIPN (OR: 0.35; 95% CI: 0.2–0.7)	[15]
*ITPA* (OR: 13.23; CI: 1.74–100.65)	Hydrolyzes inosine triphosphate and deoxyinosine triphosphate to the monophosphate nucleotide and diphosphate	rs1127354 genotype AA	Associated with the onset of grade III/IV neurological toxicity in the induction phase of the AIEOP-BFM ALL 2000 study protocol (OR: 4.61; 95% CI: 1.12–19.02)	[18]
*Cochlin*	Extracellular matrix protein found in the cochlea	rs1045466 minora allele G and rs7963521 minor allele C	Increased risk for VIPN	[20]
*CAPG*	Actin regulatory protein	rs3770102 gentoype A	A protective effect against neurotoxicity (OR: 0.1; 95% CI: 0.01–0.8)	[17]

ALL: Acute lymphoblastic leukemia; OR: Odds ratio; VCR: Vincristine; VIPN: Vincristine-induced peripheral neuropathy.

## The *CEP72* gene polymorphisms as VIPN biomarker

A promising VIPN biomarker is a genetic variant in the promoter of the *CEP72* gene. A genome-wide association study (GWAS) by Diouf and colleagues established the rs924607 TT as a VIPN risk genotype [9]. It was a prospective study of two cohorts of patients from children's oncology group (COG) and St Jude’s Hospital comprising more than 300 children. Homozygous carriers of the CEP72 rs924607 TT genotype had a higher prevalence of VIPN during the 2 years of continuation therapy, which followed induction and consolidation phases (odds ratio [OR]: 2.43; 95% CI: 1.70–3.49) and OR: 4.1; 95% CI: 1.86–9.01 in the St Jude and COG cohorts, respectively. The severity of VIPN was also higher in TT homozygotes [9]. *CEP72* encodes a protein crucial for centrosome formation. Using expression studies, Diouf *et al.* showed that TT homozygotes had significantly lower *CEP72* mRNA levels. They were able to explain this effect by demonstrating that the rs924607 T variant creates a binding site for the NKX-6.3 transcription repressor in the promoter region of the gene. Furthermore, they created pre-B ALL, T-ALL and neuroblastoma cell models and employed short hairpin RNA to impair *CEP72* gene expression. These cells were then shown to be more sensitive to VCR [9].

This study was followed by four replication studies, three of which were conducted on pediatric ALL patients and one on adult ALL patients [10–12,23]. The three studies on pediatric population produced inconsistent results, with Gutierrez-Camino *et al.* and Zgheib *et al.* reporting no association between *CEP72* rs924607 and VIPN and Wright *et al.* reporting a significantly higher risk of VIPN linked with TT genotype (OR: 3.43; 95% CI: 0.93–12.66) [10–12]. Such inconsistent results may in part be attributed to distinctive genetic backgrounds of the studied populations and the fact that Gutierrez-Camino *et al.* limited their study of VIPN to remission induction phase, while the other three studied VIPN across all phases of treatment. Some authors have also highlighted the importance of precise definitions of study end points. Namely, VIPN is most commonly diagnosed and graded using the National Cancer Institute Common Terminology Criteria for Adverse Events which classifies grade 1 as mild, grade 2 as moderate, grade 3 as serious/disabling and grade 4 as life-threatening neurotoxicity. However, these clinical scales are dependent on self-reporting of symptoms which is particularly problematic in children [24]. Alternatively, a recent study by Kavčič and colleagues has shown that VIPN can be objectively measured using electrophysiological studies [25]. Nevertheless, these procedures are uncomfortable for the children and thus unlikely to be used routinely.

A recent GWAS study found no association between the *CEP72* gene and VIPN [20]. This study was not included in our meta-analysis because genotyping data was not published.

There is also a promising ongoing clinical trial called Total Therapy XVII for Newly Diagnosed Patients With Acute Lymphoblastic Leukemia and Lymphoma sponsored by St Jude’s Research Hospital (ClinicalTrials.gov Identifier: NCT03117751) whose goal is to use strategies based on specific genomic features to improve outcomes for children with ALL and acute lymphoblastic lymphoma [26]. They are using a randomized study approach to determine whether a lower dose of VCR in patients with *CEP72* TT genotype (estimated to comprise 16% of the study population) and shorter duration of therapy in patients with *CEP72* CC or CT genotype will decrease the incidence and/or severity of VIPN. The researchers are using an unblinded design, where the treating physicians will be aware of the treatment assignments, but the investigators who evaluate neuropathy will not. The study is currently still recruiting patients [26].

In an attempt to elucidate the association between *CEP72* rs924607 and VCR toxicity we decided to conduct a meta-analysis of the results of studies on pediatric patients treated for ALL.

## Materials & methods

### Meta-analysis

#### Article selection, inclusion & exclusion criteria

To conduct the meta-analysis of data on the association between VIPN and *CEP72* rs924607 genotype TT, we searched the PubMed database for research articles by using the following keywords: ‘*CEP72* rs924607 and Vincristine’, ‘*CEP72* rs924607 and neuropathy’, ‘*CEP72* rs924607 and Vincristine andneuropathy’ as well as ‘*CEP72* rs924607 and neurotoxicity’ and ‘*CEP72* rs924607 and pediatric cancer’. Subsequently, we searched for cross-references.

Our inclusion criteria were that the study had to report the incidence of VIPN in a pediatric population treated for ALL and also include the *CEP72* rs924607 genotyping data. We excluded studies that were done on adult patients and those that were not written in the English language. We did not exclude studies based on the publication date.

From a total of six articles retrieved on PubMed, two were excluded. One was a review article that did not include genotyping data and the other a study on adult patients [3,23]. The final model comprised four studies, including 817 patients, 315 of whom had VIPN.

#### Data extraction

From the four selected articles we extracted data on the *CEP72* rs924607 genotype, incidence of VIPN, VIPN grade, duration of therapy before VIPN occurred and cumulative VCR dose received before VIPN.

Symptoms of VIPN were defined as neuropathic symptoms and sensory, motor and autonomic dysfunction (e.g., impaired tendon reflexes, balance and vibration sensation, altered gait, constipation, orthostatic hypotension). Peripheral neuropathy was graded according to the NCI Common Terminology Criteria for Adverse Events version 1.0. Patients with grades 2, 3 or 4 were considered VIPN cases. Occurrences of VIPN were the end points used for this analysis.

#### Statistical analysis

To investigate the *CEP72* rs924607 VIPN risk genotype TT, we tested for an association between this marker and VIPN using a recessive model. A one-sided p-value < 0.05 was considered statistically significant. We performed a random-effects meta-analysis using the RevMan software [27]. The OR were computed by the Mantel–Haenszel method. The association between the *CEP72* rs924607 TT genotype and VIPN was separately estimated in a subgroup of studies that assessed VIPN incidence in the continuation phases of ALL therapy. The continuation phase is defined as a treatment phase following remission induction therapy.

## Results

### Events description

This study encompasses 315 cases of VIPN from four studies (Table 2). According to data from prospective and retrospective studies a majority of VIPN cases occurred during the continuation phases of therapy. A majority of VIPN were grade 2 neuropathies (Table 3).

**Table 2.** Description of included studies.

Study	Study type	Year	Sex (%)		Mean age at ALL diagnosis in years (standard deviation/range)	Number of participants	Study finding	Ref.
			Male	Female				
Diouf *et al.*	Multiple-institutions prospective study, GWAS	2015	187 (58)	134 (42)	8.7 (0.1–23.8)	321	*CEP72* rs924607 TT genotype carries a significantly higher risk for VIPN	[9]
Gutierrez-Camino *et al.*	Multiple-institutions retrospective study	2016	81 (57)	61 (43)	5.1 (1–16)	142	No association between VIPN and *CEP72* rs924607 TT genotype	[10]
Zgheib *et al.*	Single institution retrospective study	2018	76 (57)	57 (42)	6.71 (5.01)	133	No association between VIPN and *CEP72* rs924607 TT genotype	[12]
Wright *et al.*	Nonmatched case–control study	2019	101 (81)	23 (19)	4.8 (3.3–9)^†^	224	*CEP72* rs924607 TT genotype carries a significantly higher risk for VIPN	[11]

† Median and interquartile range.

ALL: Acute lymphoblastic leukemia; VIPN: Vincristine-induced peripheral neuropathy.

**Table 3.** Vincristine-induced peripheral neuropathy case descriptions.

Study	VIPN incidence (%)	VIPN grade	Phase of therapy when VIPN occurred (%)	Mean cumulative VCR dose before VIPN mg/m^2^ (st. dev./range)	Ref.
Diouf *et al.*	86/321 (27)	Grade 2: 50	The average time to develop neuropathy was 225 days (95% CI: 169–281) in patients with the *CEP72* genotype TT, 307 days in patients with the CT/CC genotype (95% CI: 244–370)	51 (8–120)	[9]
		Grade 3: 36			
		Grade 4: 1			
Gutierrez-Camino *et al.*	36/142 (25)	NA	Only patients that developed VIPN during the induction phase were analyzed	NA	[10]
Zgheib *et al.*	26/133 (19.5)	Grade 2: 21	Induction phase: 3 (11.5)	27.91 (2.09)	[12]
		Grade 3: 5	Continuation phases: 23 (88.5)		
		Grade 4: 0			
Wright *et al.*	167/224 (74)	Grade 2: 100	Induction phase: 62 (37)	61.4 (48.0–72.0 )^†^	[11]
		Grade 3: 66	Continuation phases: 105 (63)		
		Grade 4: 1			

† Median and interquartile range.

VCR: Vincristine; VIPN: Vincristine-induced peripheral neuropathy.

### Global meta-analysis

**Figure 1.**
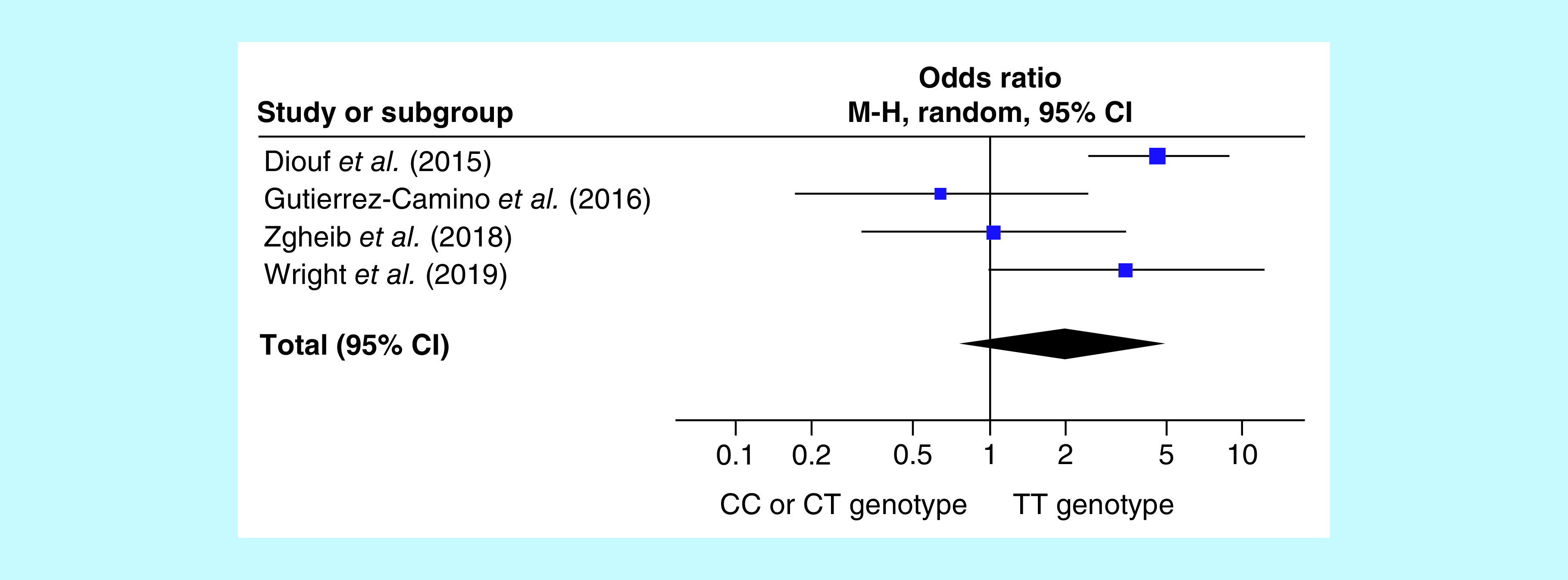
Forrest plot showing the global meta-analysis of the association between the *CEP72* rs924607 TT genotype and vincristine-induced peripheral neuropathy. (generated by RevMan [27]). The graph depicts the odds ratio of each study and its 95% CI (as blocks and lines). The diamond is the total odds ratio with its CI computed by the Mantel–Haenszel method in a random-effects meta-analysis.

When considering all included studies, the global OR for VIPN occurrence in *CEP72* rs924607 TT homozygotes was 1.99 (95% CI: 0.76–5.25; p = 0.16) (Table 4 & Figure 1).

**Table 4.** Results of the random-effects global meta-analysis testing for an association between *CEP72* rs924607 TT genotype and vincristine-induced peripheral neuropathy using a recessive model.

Study	Cases (VIPN grade ≥2)		Controls (VIPN grade <2)		Weight (%)	OR	95% CI	Heterogeneity I^2^ (p)	Test for overall effect Z (p)	Ref.
	Total	CEP72 rs924607 genotype TT	Total	CEP72 rs924607 genotype TT						
Diouf *et al.*	86	28	235	22	31.7	4.67	2.49–8.77			[9]
Gutierrez-Camino *et al.*	36	3	106	13	21.8	0.65	0.17–2.43			[10]
Zgheib *et al.*	23^†^	4	107	18	23.5	1.04	0.32–3.43			[12]
Wright *et al.*	167	27	57	3	23	3.47	1.01–11.92			[11]
Total	312	62	505	56	100	1.99	0.76–5.25	70% (0.02)	1.39 (0.16)	

† The genotyping data for three patients with VIPN was not available in the article.

OR: Odds ratio; VIPN: Vincristine-induced peripheral neuropathy.

### Subgroup analysis: continuation phase studies

**Figure 2.**
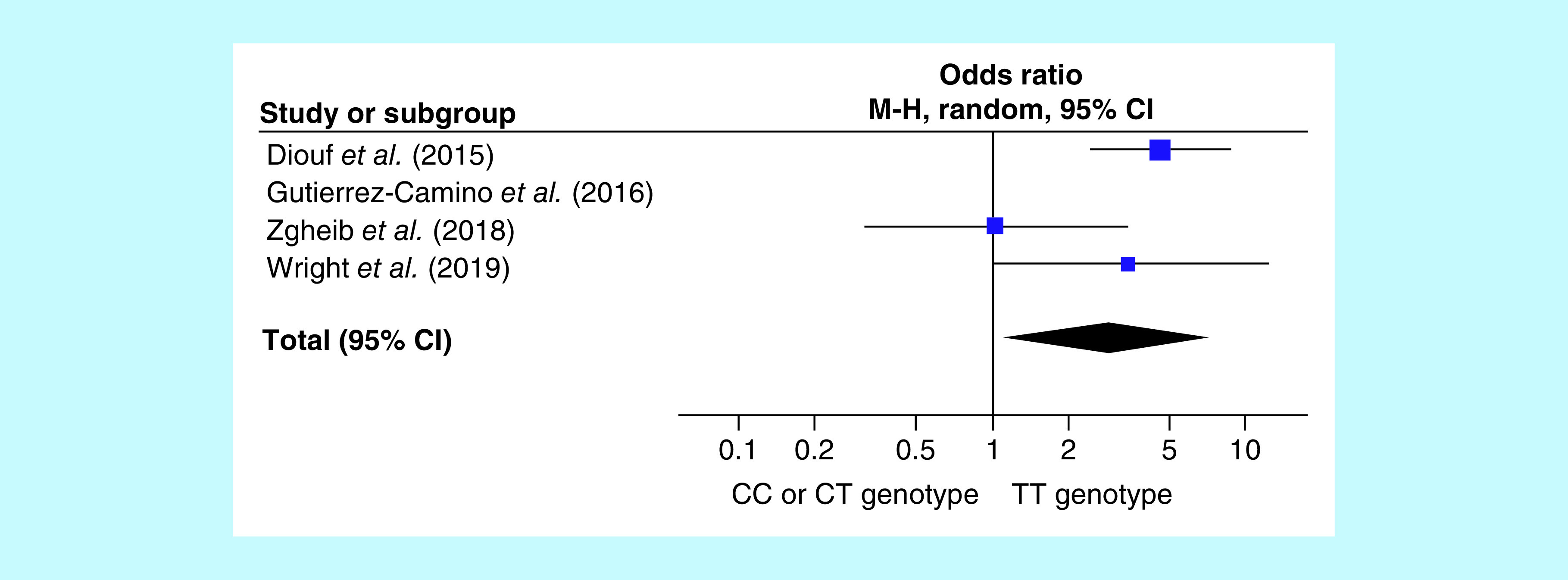
Forrest plot showing the association between the *CEP72* rs924607 TT genotype and vincristine-induced peripheral neuropathy in continuation phase studies only. (generated by RevMan [27]). The graph depicts the odds ratio of each study and its 95% CI **(as blocks and lines)**. The diamond is the total OR with its CI computed by the Mantel–Haenszel method in a random-effects meta-analysis.

When considering only continuation phases studies (excluding Gutierrez-Camino *et al.* [10]), the OR for VIPN occurrence in CEP72 rs924607 TT homozygotes is 2.82 (see Figure 2; n studies = 3; OR = 2.28; p = 0.02; 95% CI: 1.16–6.87; heterogeneity: I^2^ = 58%, p = 0.09).

## Discussion

In 2015, a study by Diouf *et al.* established *CEP72* rs924607 as a promising VIPN biomarker, by reporting a statistically significant association between *CEP72* rs924607 TT risk genotype. We analyzed the data from four such studies and revealed the association between the *CEP72* rs924607 TT genotype and VIPN in our continuation phases analysis. However, our global analysis did not confirm such association (see Table 4 & Figure 1; n studies = 4; OR: 1.99; p = 0.16; 95% CI: 0.76–5.25).

It is worth noting that the studies were conducted on populations with distinct genetic backgrounds. Both studies that confirmed the association (Diouf *et al.* and Wright *et al.*) studied the North American children while the study by Zgheib *et al.* was done in the Saudi population and the study by Gutierrez-Camino *et al.* on the Spanish population [9–12]. This may be the reason why the results differ.

However, the failure of Gutierrez-Camino *et al.* attempt to replicate the discovery study results elucidates another important consideration for further studies. Namely, it is the only study that analyzed only VIPN which occurred during the 4-week induction phase of ALL therapy [10]. Because VIPN predominantly occurs in the later phases of therapy, we decided to perform an additional meta-analysis on continuation phase studies only. In this subgroup our results show a significantly higher risk for VIPN in *CEP72* rs924607 TT homozygotes (see Figure 2; n studies = 3; OR: 2.28; p = 0.02; 95% CI: 1.16–6.87).

Wright *et al.* already published a similar meta-analysis where they reported a statistically significant association between the *CEP72* rs924607-TT genotype and VIPN both in their global analysis as well as continuation studies analysis [11]. However, they also included one study on adult ALL patients in their meta-analysis [11,23]. Therefore, the result of our meta-analysis is the first validation of this association exclusively in pediatric patients. Since we were able to replicate this finding in such a diverse pediatric ALL cohort, this pharmacogenomic biomarker could be a valuable tool for clinicians.

Although we believe our study provides valuable insight, there are some drawbacks. Even though we managed to amass a fairly large sample size, we believe including even more patients in future study cohorts would be advantageous. Small sample size and heterogeneous cohorts are common study limitations in pediatric hemato-oncology and may lead to studies producing contradictory results. A larger sample would make it easier to evaluate the effect of studying children of different ethnic and genetic backgrounds. Furthermore, the majority of the studies included in our analysis were retrospective studies, which is a limitation, as lower grade VIPN may not always be recognized from past medical records, especially in small children. A prospective study where the researchers are actively looking for drug toxicity would have been better suited for the purpose.

Therefore, we have high expectations that the Total Therapy XVII for Newly Diagnosed Patients With Acute Lymphoblastic Leukemia and Lymphoma (NCT03117751) clinical trial which is a prospective study and will hopefully further clarify the role of *CEP72* in predilection toward VIPN, and its usefulness as a biomarker in clinical practice [26]. We hope to see such studies also on other populations, not just North American, so that we can detect if the findings will be useful in European and Asian settings.

Another important future research direction for the role of *CEP72* in VCR metabolism is obtaining confirmatory evidence through functional studies. In the discovery study, Diouf and colleagues attempted to do this by impairing the expression of the gene in pre-B ALL, T-ALL and neuroblastoma cell lines and then subjecting those cells to VCR. They found that these cells were more sensitive to VCR. Furthermore, the *CEP72* promoter single nucleotide polymorphism rs924607 genotype T was shown to create a binding site for a transcriptional repressor thus reducing *CEP72* expression in human neurons and leukemia cells and increasing their sensitivity to VCR [9]. While this is an interesting finding, we need more studies to understand this relationship.

In the present study, we managed to show that *CEP72* rs924607 genotype TT is likely associated with neurotoxicity occurring during the continuation phases (after induction) of pediatric ALL treatment. However, further studies will be needed before we can implement this finding at the bedside and improve patient outcomes in the future.

## Future perspective

As high-throughput technologies are providing us with increasingly high-quality data about genomic and transcriptomic attributes of ALL patients and their response to therapy, we are bound to discover even more VIPN biomarkers. In 5–10 years, we hope to have a reliable biomarker for VIPN for the clinical setting, which will allow us to attenuate therapy with VCR in at-risk patients. This will improve both their short-term and long-term quality of life.

*CEP72* rs924607 has great potential to become such a biomarker. The Total Therapy XVII for Newly Diagnosed Patients With Acute Lymphoblastic Leukemia and Lymphoma (NCT03117751) clinical trial should provide us with additional answers on this topic. However, in the future, we hope to see more functional studies that will help us understand the mechanisms that cause VIPN and eventually prevent it.

**Summary points**

- We were unable to confirm the association between the *CEP72* rs924607 TT genotype and neurotoxicity in our global meta-analysis.
- The analysis of the continuation phase studies exposed a significantly higher risk for neuropathy in *CEP72* rs924607 TT homozygotes.
- Our meta-analysis is the first validation of the association between the *CEP72* rs924607 TT genotype and vincristine-induced peripheral neuropathy exclusively in pediatric patients.
- Because we replicated this finding in such a diverse pediatric acute lymphoblastic leukemia cohort, this pharmacogenomic biomarker could be a valuable tool for clinicians.
- Developing such biomarkers is a path toward personalized medicine.

## References

[1] Pui CH, Robison L, Look A Acute lymphoblastic leukemia. Lancet 371(9617), 1030–1043 (2008). 18358930 10.1016/S0140-6736(08)60457-2

[2] Pui CH, Evans WE Treatment of acute lymphoblastic leukemia. N. Engl. J. Med. 354(2), 166–178 (2006). 16407512 10.1056/NEJMra052603

[3] Pavlovic S, Kotur N, Stankovic B, Zukic B, Gasic V, Dokmanovic L Pharmacogenomic and pharmacotranscriptomic profiling of childhood acute lymphoblastic leukemia: paving the way to personalized treatment. Genes (Basel) 10(3), 191 (2019). 10.3390/genes10030191 PMC6471971 30832275

[4] Jordan MA, Wilson L Microtubules as a target for anticancer drugs. Nat. Rev. Cancer 4(4), 253 (2004). 15057285 10.1038/nrc1317

[5] Gidding CEM, Kellie SJ, Kamps WA, De Graaf SSN Vincristine revisited. Crit. Rev. Oncol. Hematol. 29(3), 267–287 (1999). 10226730 10.1016/s1040-8428(98)00023-7

[6] Lavoie Smith EM, Li L, Chiang C, Thomas K, Hutchinson RJ, Wells EM Patterns and severity of vincristine-induced peripheral neuropathy in children with acute lymphoblastic leukemia. J. Peripher. Nerv. Syst. 20(1), 37–46 (2015). 25977177 10.1111/jns.12114 PMC4610712

[7] Postma T, Benard B, Huijgens P, Ossenkoppele G, Heimans J Long term effects of vincristine on the peripheral nervous system. J. Neurooncol. 15(1), 23–27 (1993). 8384253 10.1007/BF01050259

[8] Lehtinen SS, Huuskonen UE, Harila-Saari AH, Tolonen U, Vainionpää LK, Lanning BM Motor nervous system impairment persists in long-term survivors of childhood acute lymphoblastic leukemia. Cancer 94(9), 2466–2473 (2002). 12015772 10.1002/cncr.10503

[9] Diouf B, Crews KR, Lew G Association of an inherited genetic variant with vincristine-related peripheral neuropathy in children with acute lymphoblastic leukemia. JAMA 313(8), 815–823 (2015). 25710658 10.1001/jama.2015.0894 PMC4377066

[10] Gutierrez-Camino A, Martin-Guerrero I, Lopez-Lopez E Lack of association of the CEP72 rs924607 TT genotype with vincristine-related peripheral neuropathy during the early phase of pediatric acute lymphoblastic leukemia treatment in a Spanish population. Pharmacogenet. Genomics 26(2), 100–102 (2016). 26618658 10.1097/FPC.0000000000000191

[11] Wright GE, Amstutz U, Drögemöller BI Pharmacogenomics of vincristine-induced peripheral neuropathy implicates pharmacokinetic and inherited neuropathy genes. Clin. Pharmacol. Ther. 105(2), 402–410 (2019). 29999516 10.1002/cpt.1179 PMC6519044

[12] Zgheib NK, Ghanem KM, Tamim H Genetic polymorphisms in candidate genes are not associated with increased vincristine-related peripheral neuropathy in Arab children treated for acute childhood leukemia: a single institution study. Pharmacogenet. Genomics 28(8), 189–195 (2018). 30119132 10.1097/FPC.0000000000000345

[13] Egbelakin A, Ferguson MJ, Macgill EA Increased risk of vincristine neurotoxicity associated with low CYP3A5 expression genotype in children with acute lymphoblastic leukemia. Pediatr. Blood Cancer 56(3), 361–367 (2011). 21225912 10.1002/pbc.22845 PMC3020258

[14] Lopez-Lopez E, Gutierrez-Camino A, Astigarraga I Vincristine pharmacokinetics pathway and neurotoxicity during early phases of treatment in pediatric acute lymphoblastic leukemia. Pharmacogenomics 17(7), 731–741 (2016). 27180762 10.2217/pgs-2016-0001

[15] Abaji R, Ceppi F, Patel S Genetic risk factors for VIPN in childhood acute lymphoblastic leukemia patients identified using whole-exome sequencing. Pharmacogenomics 19(15), 1181–1193 (2018). 30191766 10.2217/pgs-2018-0093

[16] Aplenc R, Glatfelter W, Han P CYP3A genotypes and treatment response in paediatric acute lymphoblastic leukaemia. Br. J. Haematol. 122(2), 240–244 (2003). 12846892 10.1046/j.1365-2141.2003.04430.x

[17] Ceppi F, Langlois-Pelletier C, Gagné V Polymorphisms of the vincristine pathway and response to treatment in children with childhood acute lymphoblastic leukemia. Pharmacogenomics 15(8), 1105–1116 (2014). 25084203 10.2217/pgs.14.68 PMC4443746

[18] Franca R, Rebora P, Bertorello N Pharmacogenetics and induction/consolidation therapy toxicities in acute lymphoblastic leukemia patients treated with AIEOP-BFM ALL 2000 protocol. Pharmacogenomics J. 17(1), 4 (2017). 26644204 10.1038/tpj.2015.83

[19] Guilhaumou R, Simon N, Quaranta S Population pharmacokinetics and pharmacogenetics of vincristine in paediatric patients treated for solid tumour diseases. Cancer Chemother. Pharmacol. 68(5), 1191–1198 (2011). 21404110 10.1007/s00280-010-1541-4

[20] Li L, Sajdyk T, Smith EM Genetic variants associated with vincristine-induced peripheral neuropathy in two populations of children with acute lymphoblastic leukemia. Clin. Pharmacol. Ther. 105(6), 1421–1428 (2019). 30506673 10.1002/cpt.1324 PMC6513686

[21] Dennison JB, Jones DR, Renbarger JL, Hall SD Effect of CYP3A5 expression on vincristine metabolism with human liver microsomes. J. Pharmacol. Exp. Ther. 321(2), 553–563 (2007). 17272675 10.1124/jpet.106.118471

[22] Dennison JB, Kulanthaivel P, Barbuch RJ Selective metabolism of vincristine *in vitro* by CYP3A5. Drug Metab. Dispos. 34(8), 1317–1327 (2006). 16679390 10.1124/dmd.106.009902

[23] Stock W, Diouf B, Crews KR An inherited genetic variant in CEP72 promoter predisposes to vincristine-induced peripheral neuropathy in adults with acute lymphoblastic leukemia. Clin. Pharmacol. Ther. 101(3), 391–395 (2017). 27618250 10.1002/cpt.506 PMC5320866

[24] Postma T, Heimans J, Muller M, Ossenkoppele G, Vermorken J, Aaronson N Pitfalls in grading severity of chemotherapy-induced peripheral neuropathy. Ann. Oncol. 9(7), 739–744 (1998). 9739440 10.1023/a:1008344507482

[25] Kavcic M, Koritnik B, Krzan M Electrophysiological studies to detect peripheral neuropathy in children treated with vincristine. J. Pediatr. Hematol. Oncol. 39(4), 266–271 (2017). 28375940 10.1097/MPH.0000000000000825

[26] Total therapy XVII for newly diagnosed patients with acute lymphoblastic leukemia and lymphoma (2020). https://ClinicalTrials.gov/show/NCT03117751

[27] Review manager (RevMan) [Computer program]. Version 5.3. The Nordic Cochrane Centre, The Cochrane Collaboration, Copenhagen (2014). https://training.cochrane.org/online-learning/core-software-cochrane-reviews/revman

